# Efficacy, safety, and tolerability of adjunctive brivaracetam in adult Asian patients with uncontrolled focal‐onset seizures: A phase III randomized, double‐blind, placebo‐controlled trial

**DOI:** 10.1002/epi4.12929

**Published:** 2024-04-04

**Authors:** Yushi Inoue, Somsak Tiamkao, Dong Zhou, Leonor Cabral‐Lim, Kheng Seang Lim, Shih‐Hui Lim, Jing‐Jane Tsai, Brian Moseley, Lin Wang, Weiwei Sun, Yoshinobu Hayakawa, Hiroshi Sasamoto, Tomonobu Sano, Carrie McClung, Almasa Bass

**Affiliations:** ^1^ NHO Shizuoka Institute of Epilepsy and Neurological Disorders Shizuoka Japan; ^2^ Integrated Epilepsy Research Group Khon Kaen University, Srinagarind Hospital Khon Kaen Thailand; ^3^ West China Hospital of Sichuan University Chengdu Sichuan China; ^4^ Department of Neurosciences, College of Medicine, Philippine General Hospital University of the Philippines Manila, The Health Sciences Center Manila Philippines; ^5^ Division of Neurology, Department of Medicine, Faculty of Medicine Universiti Malaya Kuala Lumpur Malaysia; ^6^ Singapore General Hospital Singapore City Singapore; ^7^ Department of Neurology National Cheng Kung University Hospital Tainan Taiwan; ^8^ UCB Pharma Morrisville North Carolina USA; ^9^ UCB Pharma Shanghai China; ^10^ UCB Pharma Tokyo Japan

**Keywords:** antiseizure medication, Asian, clinical trial, concomitant medication, focal‐onset epilepsy

## Abstract

**Objective:**

Evaluate efficacy, safety, and tolerability of adjunctive brivaracetam (BRV) in adult Asian patients with focal‐onset seizures (FOS).

**Methods:**

Phase III, randomized, double‐blind, placebo‐controlled study (EP0083; NCT03083665) evaluating BRV 50 mg/day and 200 mg/day in patients (≥16–80 years) with FOS with/without secondary generalization (focal to bilateral tonic–clonic seizures) despite current treatment with 1 or 2 concomitant antiseizure medications. Following an 8‐week baseline, patients were randomized 1:1:1 to placebo, BRV 50 mg/day, or BRV 200 mg/day, and entered a 12‐week treatment period. Efficacy outcomes: percent reduction over placebo in 28‐day FOS frequency (primary); 50% responder rate in FOS frequency; median percent reduction in FOS frequency from baseline; seizure freedom during treatment period (secondary). Primary safety endpoints: incidences of treatment‐emergent adverse events (TEAEs); TEAEs leading to discontinuation; serious TEAEs.

**Results:**

In this study, 448/449 randomized patients (mean age, 34.5 years; 53.8% female) received ≥1 dose of study medication (placebo/BRV 50 mg/BRV 200 mg/day: *n* = 149/151/148). Percent reduction over placebo in 28‐day adjusted FOS frequency was 24.5% (*p* = 0.0005) and 33.4% (*p* < 0.0001) with BRV 50 mg/day and 200 mg/day, respectively, 50% responder rate was 19.0%, 41.1%, and 49.3% with placebo, BRV 50 mg/day, and BRV 200 mg/day, respectively (*p* < 0.0001 for both BRV groups vs. placebo). Median percent reduction in FOS frequency from baseline was 21.3%/38.9%/46.7% in patients on placebo/BRV 50 mg/BRV 200 mg/day, respectively. Overall, 0, 7 (4.6%), and 10 (6.8%) patients were classified as seizure‐free during the treatment period on placebo, BRV 50 mg/day, and BRV 200 mg/day, respectively (*p* = 0.0146/*p* = 0.0017 for BRV 50 mg/200 mg/day vs. placebo, respectively). TEAE incidences were similar between patients on placebo (58.4%) and all patients receiving BRV (58.5%); TEAE incidences for BRV 50 mg/day and BRV 200 mg/day were 57.0% and 60.1%, respectively. Overall, 0.7% of patients on placebo and 2.0% of all patients on BRV reported serious TEAEs (incidences for BRV 50 mg/day and BRV 200 mg/day were 1.3% and 2.7%, respectively), 20.1% of patients on placebo and 33.1% of all patients on BRV reported drug‐related TEAEs (incidences for BRV 50 mg/day and BRV 200 mg/day were 26.5% and 39.9%, respectively), and 4.7% of patients on placebo and 3.0% of all patients on BRV discontinued due to TEAEs (discontinuation incidences for BRV 50 mg/day and BRV 200 mg/day were 2.6% and 3.4%, respectively).

**Significance:**

Adjunctive BRV was efficacious and well tolerated in adult Asian patients with FOS. Efficacy and safety profiles were consistent with BRV studies in predominantly non‐Asian populations.

**Plain Language Summary:**

Brivaracetam is used to treat partial or focal seizures in people with epilepsy. Most studies with brivaracetam tablets have involved people from non‐Asian racial backgrounds. In this study, 449 Asian adults with epilepsy took part. One third took 50 mg of brivaracetam, one third took 200 mg of brivaracetam, and one third took a placebo each day for 12 weeks. On average, those who took brivaracetam had fewer seizures than those given the placebo. Most of the side effects were mild and the number and type of side effects seen were as expected for this medication.

## Graphical abstract

This study aimed to evaluate the efficacy, safety, and tolerability of adjunctive brivaracetam in adult Asian patients with focal‐onset seizures. The efficacy, safety, and tolerability profiles were consistent with brivaracetam studies in predominantly non‐Asian populations, demonstrating that adjunctive brivaracetam was efficacious and well‐tolerated in adult Asian patients with focal‐onset seizures.

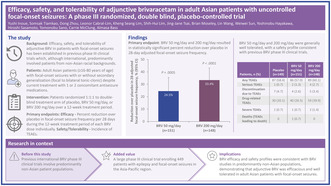

**Key points**

- Phase III, randomized, double‐blind, placebo‐controlled study of brivaracetam (BRV) in adult Asian patients with focal‐onset seizures.
- Percent reduction over placebo in 28‐day adjusted focal seizure frequency was 24.5% and 33.4% with BRV 50 and 200 mg/day, respectively.
- 50% responder rate was 19.0%, 41.1%, and 49.3% with placebo, BRV 50 mg/day, and BRV 200 mg/day, respectively.
- Incidences of treatment‐emergent adverse events were 58.4%, 57.0%, and 60.1% for placebo, BRV 50 mg/day, and BRV 200 mg/day, respectively.
- The above data demonstrate that BRV was efficacious and well tolerated in Asian populations.

## 1 INTRODUCTION

Epilepsy is treated pharmacologically with antiseizure medications (ASMs); however, up to 30% of patients cannot achieve seizure freedom with currently available ASMs.^1^, ^2^ The overall effectiveness of any ASM is determined not only by its efficacy but also by its safety and tolerability.^3^ A successful treatment requires consideration of multiple factors, such as the antiseizure effects, adverse effects of the ASM, and the patient's individual susceptibility to these adverse effects.^4^ Lack of tolerability of an ASM can be a driver for nonadherence,^5^ which may lead to breakthrough seizures.

Brivaracetam (BRV) is indicated for adjunctive therapy of focal‐onset (partial‐onset) seizures in patients aged ≥2 years in the European Union^6^ and for mono‐ and adjunctive therapy in patients aged ≥1 month in the United States.^7^ In the Asia‐Pacific region, BRV is approved in Taiwan for mono‐ and adjunctive therapy of focal‐onset seizures in patients aged ≥4 years (intravenous formulation approved in adults ≥16 years), and in Hong Kong and the Republic of Korea for adjunctive therapy of focal‐onset seizures in patients aged ≥16 years (BRV not launched in the Republic of Korea).

The efficacy, safety, and tolerability of adjunctive BRV in patients with focal‐onset seizures have been established in phase III international clinical trials^8^, ^9^, ^10^ and their corresponding long‐term follow‐up (LTFU) studies.^11^, ^12^, ^13^ A post hoc pooled analysis of data from the phase III trials supported efficacy, safety, and tolerability of adjunctive BRV in a large population of patients with focal‐onset seizures.^14^ Effectiveness and tolerability of BRV have also been demonstrated in a number of real‐world studies.^15^, ^16^, ^17^

Although a phase I study in Japanese participants showed that no BRV dose modification is required for Asian patients,^18^ BRV clinical data for the Asian population are rather limited since the international phase III trials predominantly recruited patients from a non‐Asian racial background.^8^, ^9^, ^10^

BRV binds to the synaptic vesicle glycoprotein 2A (SV2A) receptor site with a high affinity and selectivity.^19^, ^20^ Previous treatment failure with levetiracetam (LEV), which also binds to the SV2A receptor site, but with less affinity vs. BRV, has been shown not to preclude BRV use in patients with epilepsy.^9^, ^21^ BRV has also shown efficacy, safety, and tolerability in combination with specific concomitant ASMs, including carbamazepine (CBZ), lamotrigine (LTG), oxcarbazepine (OXC), and valproate (VPA).^22^, ^23^, ^24^

The current phase III EP0083 study aimed to evaluate the efficacy, safety, and tolerability of BRV in Asian patients with focal‐onset seizures (aged ≥16–80 years). Effects of previous LEV exposure and common concomitant ASMs at study entry on key efficacy and tolerability outcomes were also explored.

## 2 METHODS

### 2.1 Study design

EP0083 (ClinicalTrials.gov: NCT03083665) was a phase III, randomized, double‐blind, placebo‐controlled, multicenter, parallel‐group, therapeutic confirmatory study evaluating two dosages of BRV (50 mg/day, 200 mg/day) as adjunctive treatment in Asian patients (aged ≥16–80 years) with focal‐onset seizures with or without secondary generalization despite current treatment with 1 or 2 permitted concomitant ASMs.

Overall, there were 94 participating study sites located in Japan (40), China Mainland (23), Malaysia (8), the Philippines (8), Thailand (8), Taiwan (5), and Singapore (2). A list of investigators is included in Appendix S1.

This study was conducted in compliance with International Council for Harmonisation Good Clinical Practice (GCP), Japan's GCP, and the Declaration of Helsinki. The study protocol, amendments, and patient informed consent were approved by local institutional review boards/independent ethics committees, as defined in local regulations. Patients provided informed consent or had a legally authorized representative sign the informed consent on their behalf before completing any study‐related procedures.

### 2.2 Patient population

Patients were aged ≥16–80 years with a history of focal‐onset seizures with or without secondary generalization. Inclusion criteria included patients having at least eight focal‐onset seizures (in accordance with 1981 International League Against Epilepsy [ILAE] classification and without discrepancies with 2017 ILAE classification^25^, ^26^) during the 8‐week baseline period, with at least two focal‐onset seizures during each 4‐week interval of the baseline period; patients having at least two focal‐onset seizures, with or without secondary generalization, per month during the 3 months before screening (visit 1); and patient's condition being uncontrolled with treatment with ≤2 ASMs that were at a stable/optimal dose for ≥4 weeks before visit 1 and expected to remain stable throughout the study period. Vagal nerve stimulation was permitted and counted as a concomitant ASM. Patients with previous LEV use (LEV taken and stopped >90 days before screening [visit 1]) were enrolled such that the ratio of previous LEV users was maintained at a maximum of 30% of the whole study population.

Exclusion criteria included patients having a history or presence of status epilepticus during the year preceding visit 1 or during baseline and patients currently treated with LEV or having taken LEV within 90 days before visit 1. Full inclusion and exclusion criteria are described in Appendix S2.

### 2.3 Treatment schedule

Total duration of the study was 26 weeks (maximum 16 weeks of exposure to BRV), comprising an 8‐week prospective baseline period and a 12‐week treatment period. For patients not entering a subsequent LTFU study (ClinicalTrials.gov: NCT03250377; EP0085) or managed access program (MAP), the treatment period was followed by a 4‐week down‐titration period and a 2‐week study drug−free period. For patients participating in the LTFU study or MAP, the treatment period was followed by a 2‐week transition period. If a MAP was not ready at the end of the transition period, patients converted to the open‐label temporary period for providing BRV (Figure S1). After the 8‐week baseline period, patients were randomized to three treatment arms in a central randomization (random permuted blocks) 1:1:1 ratio (placebo comparator:BRV 50 mg/day:BRV 200 mg/day) and entered the double‐blind treatment period (visits 3–7). The BRV 50 mg/day and BRV 200 mg/day treatment arms were chosen based on the minimum and maximum BRV daily doses approved worldwide. BRV was initiated without titration and patients received assigned randomization doses from the first day of treatment. BRV was administered orally twice daily in equally divided doses. Oral film‐coated tablets of BRV 25 mg, BRV 50 mg, and matching placebo tablets were used. Stratification for LEV status (LEV naïve vs. previous LEV use), number of ASMs previously used (≤2 vs. >2 ASMs), and country at study entry ensured balance across treatment groups (placebo, BRV 50 mg/day, BRV 200 mg/day) within each combination of stratification levels.

### 2.4 Endpoints

Efficacy outcomes were evaluated over the 12‐week treatment period. The primary efficacy endpoint was percent reduction over placebo in 28‐day focal‐onset seizure frequency of each BRV dose individually. Secondary efficacy endpoints were 50% responder rate based on percent reduction in 28‐day focal‐onset seizure frequency from baseline; median percent reduction in 28‐day focal‐onset seizure frequency from baseline; and seizure freedom (all seizures; patients were defined as seizure free if they had no missing diary days, no reported seizures during the treatment period, and had completed the treatment period).

Primary safety endpoints were incidence of treatment‐emergent adverse events (TEAEs; Medical Dictionary for Regulatory Activities, version 18.1), incidence of TEAEs leading to study discontinuation, and incidence of treatment‐emergent serious adverse events (AEs); TEAEs from all study periods except the open‐label temporary period are reported. Other safety endpoints included changes in clinical laboratory tests parameters, vital signs, and body weight; electrocardiogram (ECG) parameters and findings; physical and neurological examinations; assessment of mental and psychiatric status; and assessment of suicidality. Clinically significant new or worsened abnormalities in physical or neurological examinations or psychiatric and mental status were reported as AEs.

The pharmacokinetic endpoint was plasma concentration of BRV.

### 2.5 Subgroup and post hoc analyses

A prespecified subgroup analysis was conducted for key efficacy outcomes for LEV‐naïve patients and patients with previous LEV use at study entry. The analysis was not stratified further by reason for previous LEV failure (lack of efficacy versus tolerability) in patients with previous LEV use at study entry. Baseline patient demographics, epilepsy characteristics, and a tolerability overview by LEV status were generated post hoc. A post hoc subgroup analysis was also conducted for key efficacy and tolerability outcomes, baseline patient demographics, and epilepsy characteristics across the most common (defined as ≥30 patients in any BRV subgroup) concomitant ASMs subgroups (VPA, CBZ, LTG) used at study entry (independent of number of concomitant ASMs).

### 2.6 Statistical analyses

A sample size of 148 patients per treatment group (444 total) was planned for this study. This sample size provided 80% power to simultaneously detect differences between the two BRV treatment groups (50 mg/day, 200 mg/day) and the placebo group at the one‐sided 0.025 significance level. Further information on sample size calculations is provided in Appendix S2.

Analysis sets were defined as follows: the randomized set (RS) included all patients randomized to a treatment arm; the safety set (SS) included all patients from the RS who received at least one dose of study drug; the full analysis set (FAS) included all patients from the SS who had at least one post‐baseline seizure daily record card data entry during the treatment period; and the pharmacokinetic per‐protocol set (PK‐PPS) included all patients who took at least one dose of BRV and for whom at least one valid BRV plasma concentration time and dosing information were available.

Primary analyses, based on analysis of covariance (ANCOVA) with log‐transformed [log(*x* + 1)] treatment period 28‐day adjusted focal seizure frequency as the outcome, included an effect for treatment, an effect for country, and an effect for the four combinations of actual stratification levels for LEV status and number of previous ASMs. Lower enrolling countries were pooled to minimize the risk of issues due to data sparsity. Primary analyses were also adjusted for log‐transformed (log[*x* + 1]) 28‐day adjusted baseline focal seizure frequency as a continuous covariate. Adjustment for this covariate accounted for any imbalance across treatment groups with respect to baseline focal seizure frequency. Statistical testing was based on the comparison of each BRV treatment group (BRV 50 mg/day, 200 mg/day) with placebo with control of overall type I error rate based on the Hochberg multiple comparisons procedure. During conduct of randomization, stratification levels for some patients were entered incorrectly into the interactive voice response system (IVRS). However, the stratification data recorded in the eCRF were correct, with stratification levels balanced among the three treatment groups. Number of patients for each stratification factor per IVRS and eCRF is shown in Appendix S2. A sensitivity analysis using a similar ANCOVA model was performed according to the IVRS stratification level to assess the impact of this error on the primary efficacy variable. Statistical analyses were carried out using SAS® version 9.3.

All subgroup and post hoc analyses were descriptive and no statistical comparisons were performed.

## 3 RESULTS

### 3.1 Patient disposition and discontinuation

Between August 22, 2017 and June 30, 2022, 571 patients were screened at 94 sites, with 122 screen failures. In total, 449 patients were randomized into the treatment arms and included in the current analyses (RS: *N* = 449; SS: *N* = 448; FAS: *N* = 446; PK‐PPS: *N* = 296). Most patients (≥92.6%) in each treatment arm completed the study. Overall, 11 (7.4%), 5 (3.3%), and 8 (5.4%) patients in the placebo, BRV 50 mg/day, and BRV 200 mg/day treatment groups, respectively, discontinued the study. The most common reason for study discontinuation in each treatment group was due to AEs (5 [3.4%], 4 [2.6%], and 5 [3.4%] patients in the placebo, BRV 50 mg/day, and BRV 200 mg/day treatment groups, respectively; Table S1).

### 3.2 Overall population

#### 3.2.1 Baseline demographics, epilepsy characteristics, and BRV dosing

At baseline, patients had a mean (standard deviation [SD]) age of 34.5 (13.0) years and 53.8% were female (Table 1). The mean (SD) duration of epilepsy was 16.6 (12.2) years and age at onset was 18.4 (13.2) years. The number of patients with 0, 1, 2, 3, 4, and ≥5 previous ASMs (previous ASMs were ASMs that were taken and discontinued at any time before study entry) were 131 (29.4%), 101 (22.6%), 63 (14.1%), 45 (10.1%), 35 (7.8%), and 71 (15.9%), respectively (Table 2). Patient baseline demographics were similar across treatment arms. In all patients exposed to BRV (*N* = 299) during the treatment period, the median duration of exposure was 85.0 (range, 1–96) days; for patients on placebo, the median duration of exposure was 85.0 (range, 7–92) days.

**TABLE 1:** Baseline demographics (SS).

	Placebo (*n* = 149)	BRV 50 mg/day (*n* = 151)	BRV 200 mg/day (*n* = 148)	BRV all (*n* = 299)	All patients (*N* = 448)
Age, mean (SD), years	34.5 (13.2)	33.8 (12.6)	35.2 (13.2)	34.5 (12.9)	34.5 (13.0)
Age, range, years	16–80	16–68	16–78	16–78	16–80
Female, *n* (%)	82 (55.0)	76 (50.3)	83 (56.1)	159 (53.2)	241 (53.8)
Weight, mean (SD), kg	63.2 (14.0)	64.3 (14.9)	61.3 (14.8)	62.8 (14.9)	62.9 (14.6)
Height, mean (SD), cm	162.2 (8.3)	162.5 (8.5)	162.0 (9.0)	162.3 (8.8)	162.3 (8.6)
Racial group, *n* (%)					
Asian	149 (100)	151 (100)	148 (100)	299 (100)	448 (100)
Country, *n* (%)					
Thailand	49 (32.9)	48 (31.8)	48 (32.4)	96 (32.1)	145 (32.4)
Japan	34 (22.8)	31 (20.5)	32 (21.6)	63 (21.1)	97 (21.7)
China Mainland	27 (18.1)	31 (20.5)	28 (18.9)	59 (19.7)	86 (19.2)
Philippines	20 (13.4)	20 (13.2)	22 (14.9)	42 (14.0)	62 (13.8)
Malaysia	15 (10.1)	16 (10.6)	16 (10.8)	32 (10.7)	47 (10.5)
Singapore	1 (0.7)	4 (2.6)	1 (0.7)	5 (1.7)	6 (1.3)
Taiwan	3 (2.0)	1 (0.7)	1 (0.7)	2 (0.7)	5 (1.1)

Abbreviations: BRV, brivaracetam; SD, standard deviation; SS, safety set.

**TABLE 2:** Epilepsy characteristics and ASM use (FAS).

	Placebo (*n* = 147)	BRV 50 mg/day (*n* = 151)	BRV 200 mg/day (*n* = 148)	BRV all (*n* = 299)	All patients (*N* = 446)
Duration of epilepsy, mean (SD), years	17.2 (11.8)	17.1 (12.2)^a^	15.4 (12.6)	16.2 (12.4)^b^	16.6 (12.2)^c^
Age at onset of epilepsy, mean (SD), years	17.7 (13.9)	17.2 (11.8)^a^	20.3 (13.7)	18.7 (12.9)^b^	18.4 (13.2)^c^
Baseline focal‐onset seizure frequency/28 days, median (Q1, Q3)	9.8 (5.5, 22.2)	9.0 (5.1, 17.5)	7.8 (5.0, 16.5)	8.2 (5.0, 16.6)	8.5 (5.1, 17.6)
Seizure classification,^d^ *n* (%)					
Any focal‐onset seizures	147 (100)	151 (100)	148 (100)	299 (100)	446 (100)
Focal aware	82 (55.8)	72 (47.7)	73 (49.3)	145 (48.5)	227 (50.9)
Focal impaired awareness	129 (87.8)	125 (82.8)	119 (80.4)	244 (81.6)	373 (83.6)
Focal to bilateral tonic–clonic	88 (59.9)	91 (60.3)	81 (54.7)	172 (57.5)	260 (58.3)
Any generalized seizures	10 (6.8)	3 (2.0)	7 (4.7)	10 (3.3)	20 (4.5)
Unclassified epileptic seizures	1 (0.7)	0	1 (0.7)	1 (0.3)	2 (0.4)
Number of previous ASMs,^e^ *n* (%)					
0	39 (26.5)	44 (29.1)	48 (32.4)	92 (30.8)	131 (29.4)
1	36 (24.5)	33 (21.9)	32 (21.6)	65 (21.7)	101 (22.6)
2	18 (12.2)	24 (15.9)	21 (14.2)	45 (15.1)	63 (14.1)
3	16 (10.9)	16 (10.6)	13 (8.8)	29 (9.7)	45 (10.1)
4	14 (9.5)	11 (7.3)	10 (6.8)	21 (7.0)	35 (7.8)
≥5	24 (16.3)	23 (15.2)	24 (16.2)	47 (15.7)	71 (15.9)
Previous LEV use	40 (27.2)	42 (27.8)	35 (23.6)	77 (25.8)	117 (26.2)
ASMs taken at study entry,^f^ by ≥5% of all patients, *n* (%)					
Valproate	44 (29.9)	68 (45.0)	65 (43.9)	133 (44.5)	177 (39.7)
Carbamazepine	50 (34.0)	46 (30.5)	40 (27.0)	86 (28.8)	136 (30.5)
Lamotrigine	32 (21.8)	32 (21.2)	36 (24.3)	68 (22.7)	100 (22.4)
Phenytoin	19 (12.9)	25 (16.6)	27 (18.2)	52 (17.4)	71 (15.9)
Topiramate	15 (10.2)	16 (10.6)	16 (10.8)	32 (10.7)	47 (10.5)
Phenobarbital	18 (12.2)	16 (10.6)	8 (5.4)	24 (8.0)	42 (9.4)
Lacosamide	12 (8.2)	16 (10.6)	13 (8.8)	29 (9.7)	41 (9.2)
Oxcarbazepine	10 (6.8)	10 (6.6)	18 (12.2)	28 (9.4)	38 (8.5)
Perampanel	12 (8.2)	6 (4.0)	7 (4.7)	13 (4.3)	25 (5.6)

Abbreviations: ASM, antiseizure medication; BRV, brivaracetam; eCRF, electronic case report form; FAS, full analysis set; SD, standard deviation.

^a^ *n* = 150.

^b^ *n* = 298.

^c^ *n* = 445.

^d^ Seizures experienced at any time before study entry are summarized.

^e^ Previous ASMs are ASMs taken at any time before study entry and discontinued before study entry and are reported ASMs in the history of previous ASMs page of eCRF.

^f^ World Health Organization Drug Dictionary SEP/2017 Preferred Drug Name.

#### 3.2.2 Efficacy

**FIGURE 1:**
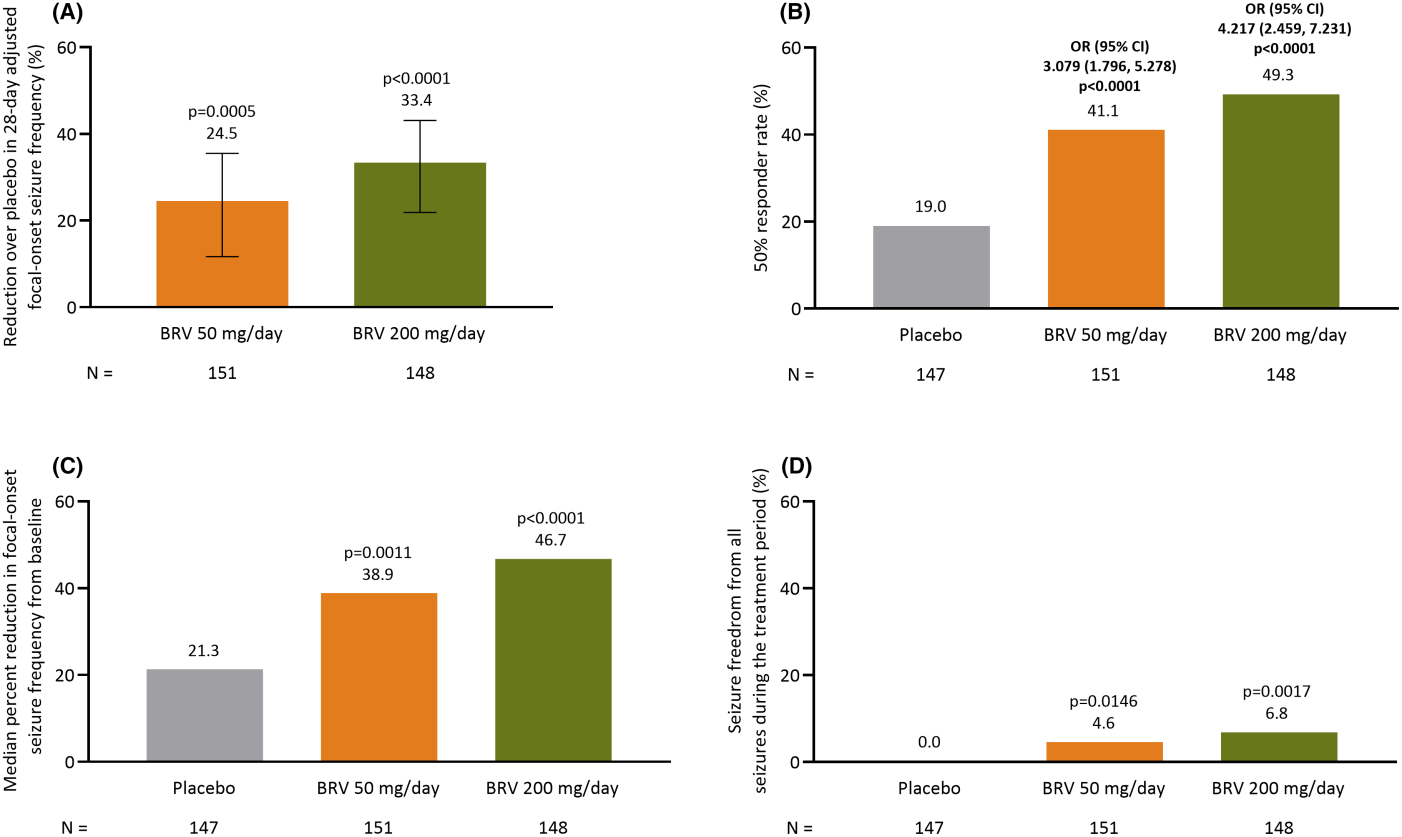
Efficacy analysis for the overall study population: (A) percent reduction over placebo in 28‐day adjusted focal‐onset seizure frequency^a,b^, (B) 50% responder rate in focal‐onset seizure frequency from baseline^c^, (C) median percent reduction in focal‐onset seizure frequency from baseline^b^, and (D) seizure freedom from all seizure types during the treatment period^b^ (FAS). ^a^Error bars represent 95% CIs. ^b^ *p*‐values represent BRV versus placebo. ^c^ *p*‐values represent odds ratio (BRV versus placebo). BRV, brivaracetam; CI, confidence interval; FAS, full analysis set; OR, odds ratio.

During baseline, median 28‐day focal‐onset seizure frequency in the placebo, BRV 50 mg/day, and BRV 200 mg/day groups was 9.8, 9.0, and 7.8, respectively; this decreased to 7.2, 5.9, and 4.2, respectively, during the treatment period. Percent reductions over placebo in 28‐day adjusted focal‐onset seizure frequency were 24.5% and 33.4% for BRV 50 mg/day and BRV 200 mg/day, respectively. These reductions were statistically significant for both BRV groups (*p* = 0.0005 and *p* < 0.0001 respectively; Figure 1A). Outcomes of the sensitivity analysis on the primary efficacy variable yielded similar results of statistically significant reductions of both BRV treatment groups over placebo (Table S2).

The 50% responder rate for focal‐onset seizure frequency was 19.0%, 41.1%, and 49.3% in the placebo, BRV 50 mg/day, and BRV 200 mg/day groups, respectively. The odds ratio (BRV vs. placebo) for the 50% responder rate in the BRV 50 mg/day group was 3.079 (95% confidence interval [CI], 1.796–5.278 [*p* < 0.0001]) and in the BRV 200 mg/day group was 4.217 (95% CI, 2.459–7.231 [*p* < 0.0001] Figure 1B).

Median percent reduction in focal‐onset seizure frequency from baseline over the 12‐week treatment period was 21.3%, 38.9%, and 46.7% in patients randomized to placebo, BRV 50 mg/day, and BRV 200 mg/day, respectively (Figure 1C); median differences versus placebo for the BRV 50 mg/day and BRV 200 mg/day groups were 16.0% and 24.4%, respectively (*p* = 0.0011 and *p* < 0.0001, respectively).

For BRV 50 mg/day and BRV 200 mg/day groups, 7 (4.6%) and 10 (6.8%) patients were classified as seizure free (all seizure types) during the treatment period, respectively. No patients on placebo were classified as seizure free. Numbers of seizure‐free patients in the BRV 50 mg/day and BRV 200 mg/day groups were significantly greater than the placebo group (*p* = 0.0146 and *p* = 0.0017 respectively; Figure 1D).

#### 3.2.3 Safety and tolerability

For the primary safety endpoints, during all study periods (except the open‐label temporary period), 175/299 (58.5%) patients receiving any dose of BRV and 87/149 (58.4%) patients receiving placebo reported TEAEs (Table 3), and of these, most were mild (145/175 [82.9%] and 72/87 [82.8%], respectively). Overall, the most common (≥10%) TEAEs by preferred term in all patients receiving BRV were somnolence (14.4%) and dizziness (12.7%); incidences of somnolence and dizziness in patients receiving placebo were 8.1% and 4.0%, respectively. Serious TEAEs were reported in 6 (2.0%) patients receiving BRV and 1 (0.7%) patient receiving placebo. Discontinuations due to TEAEs were reported in 9 (3.0%) patients receiving BRV and 7 (4.7%) patients receiving placebo. Drug‐related TEAEs were reported in 99 (33.1%) patients receiving BRV and 30 (20.1%) patients receiving placebo; incidences of drug‐related TEAEs were numerically higher in the BRV 200 mg/day group than the BRV 50 mg/day group (59 [39.9%] vs. 40 [26.5%] patients, respectively). One death due to drowning was reported during the study in a patient randomized to BRV 50 mg/day (considered unrelated to BRV by the investigator).

**TABLE 3:** Incidence of TEAEs (SS).^a^

Patients, *n* (%)	Placebo (*n* = 149)	BRV 50 mg/day (*n* = 151)	BRV 200 mg/day (*n* = 148)	BRV all (*n* = 299)
Any TEAEs	87 (58.4)	86 (57.0)	89 (60.1)	175 (58.5)
Serious TEAEs	1 (0.7)	2 (1.3)	4 (2.7)	6 (2.0)
Discontinuation due to TEAEs	7 (4.7)	4 (2.6)	5 (3.4)	9 (3.0)
Drug‐related TEAEs	30 (20.1)	40 (26.5)	59 (39.9)	99 (33.1)
Severe TEAEs	1 (0.7)	1 (0.7)	2 (1.4)	3 (1.0)
Deaths (TEAEs leading to death)	0	1 (0.7)	0	1 (0.3)
TEAEs^a^ ^,^ ^b^ occurring in ≥3% of all patients receiving BRV				
Somnolence	12 (8.1)	15 (9.9)	28 (18.9)	43 (14.4)
Dizziness	6 (4.0)	17 (11.3)	21 (14.2)	38 (12.7)
Headache	11 (7.4)	11 (7.3)	7 (4.7)	18 (6.0)
URTI	7 (4.7)	10 (6.6)	8 (5.4)	18 (6.0)
Nasopharyngitis	10 (6.7)	7 (4.6)	10 (6.8)	17 (5.7)
Weight decreased	2 (1.3)	5 (3.3)	4 (2.7)	9 (3.0)
TEAEs^a^ ^,^ ^b^ leading to discontinuation in ≥1% of patients in any treatment group				
Dizziness	2 (1.3)	1 (0.7)	1 (0.7)	2 (0.7)
Seizure	3 (2.0)	0	1 (0.7)	1 (0.3)

Abbreviations: BRV, brivaracetam; SS, safety set; TEAE, treatment‐emergent adverse event; URTI, upper respiratory tract infection.

^a^ Inclusive of all study periods except open‐label temporary period.

^b^ Preferred term (Medical Dictionary for Regulatory Activities, version 18.1).

Psychiatric TEAEs were reported in 16 (5.4%) patients receiving BRV and in 6 (4.0%) patients receiving placebo. For the most common psychiatric TEAEs by preferred term, insomnia was reported in 5 (1.7%) patients receiving BRV and 3 (2.0%) patients receiving placebo, depression was reported in 3 (1.0%) and 0 patients, and irritability was reported in 2 (0.7%) and 2 (1.3%) patients, respectively. There were no events of suicide attempt, and TEAEs of suicidal ideation were reported by 1 (0.3%) patient receiving BRV and 1 (0.7%) patient receiving placebo. Five (1.7%) patients who received BRV discontinued due to psychiatric TEAEs; by preferred term, these psychiatric TEAEs were depression (2 [0.7%] patients), irritability, postictal psychosis, and suicidal ideation (1 [0.3%] patient each). No patients on placebo discontinued due to psychiatric TEAEs.

Most of the psychiatric and mental status findings that occurred in any treatment group during the treatment period were also present at baseline. Of 3 patients who had abnormal psychiatric symptoms at the early discontinuation visit that were not present at baseline, 1 patient received BRV 50 mg/day (and reported depression) and 2 patients received BRV 200 mg/day (1 reported depression and 1 reported irritability).

There were no clinically meaningful differences from baseline or between treatment groups in laboratory parameters, vital signs, ECG evaluations, physical examinations, or neurological examinations.

### 3.3 Pharmacokinetics

Geometric mean BRV plasma concentrations per blood collection time window (>0–4, >4–8, >8 h after dosing) were consistent across the treatment period and were higher in the BRV 200 mg/day versus BRV 50 mg/day group (Table S3). Geometric mean dose‐normalized BRV plasma concentrations were comparable between BRV treatment groups Table S4, supporting the dose‐proportional pharmacokinetics of BRV (Table S3).

### 3.4 Subgroup analysis by LEV status

#### 3.4.1 Baseline demographics and epilepsy characteristics

In total, 329 (73.8%) patients were LEV naïve, and 117 (26.2%) patients had previous exposure to LEV (Table 2). In the previous LEV‐use subgroup, mean (SD) age was 33.5 (12.5) years and 64.1% were female, whereas in the LEV‐naïve subgroup, mean (SD) age was 34.8 (13.1) years and 50.2% were female. Patients with previous LEV‐use versus LEV‐naïve patients had numerically higher 28‐day baseline focal‐onset seizure frequency (10.5 vs. 7.5), higher median number of previous ASMs (4.0 vs. 1.0) and included a higher proportion of patients on two concomitant ASMs at study drug initiation (76.9% vs. 58.1%). The most common concomitant ASMs in both subgroups were VPA, CBZ, and LTG (LEV naïve, 41.3%, 28.6%, and 20.4%, respectively; previous LEV use, 35.0%, 35.9%, and 28.2%, respectively).

#### 3.4.2 Efficacy

**FIGURE 2:**
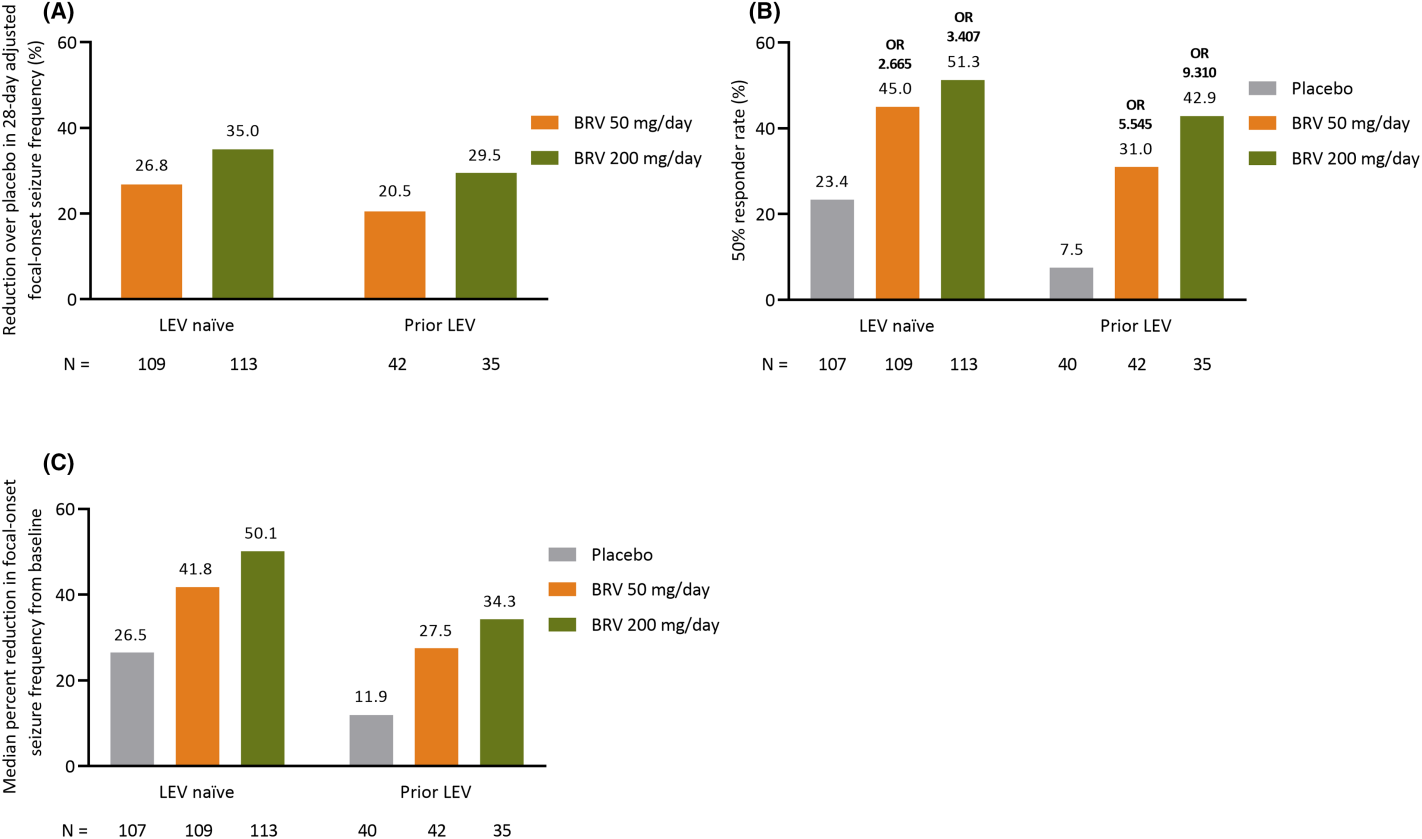
Efficacy for the subgroup analysis by LEV status: (A) percent reduction over placebo in 28‐day adjusted focal‐onset seizure frequency, (B) 50% responder rate in focal‐onset seizure frequency from baseline^a^, and (C) median percent reduction in focal‐onset seizure frequency from baseline (FAS). ^a^Odds ratio represent BRV versus placebo. BRV, brivaracetam; FAS, full analysis set; LEV, levetiracetam; OR, odds ratio.

BRV provided clinically relevant percent reductions over placebo in 28‐day adjusted focal‐onset seizure frequency (Figure 2A), greater 50% responder rate for focal‐onset seizure frequency (Figure 2B), and greater median reductions from baseline in 28‐day adjusted focal‐onset seizure frequency than placebo, irrespective of LEV status (Figure 2C). However, a numerically greater response was observed in the LEV‐naïve group for all these variables.

#### 3.4.3 Safety and tolerability

In the LEV‐naïve subgroup, TEAE incidences for all patients receiving BRV (*n* = 222) and patients receiving placebo (*n* = 109) were 57.2% and 53.2%, respectively (Table S5). Incidences of drug‐related TEAEs were 32.4% and 14.7%, discontinuations due to TEAEs were 2.3% and 3.7%, serious TEAEs were 1.8% and 0.9%, and severe TEAEs were 0.9% and 0.9% for all patients receiving BRV and patients receiving placebo, respectively.

In the previous LEV‐use subgroup, TEAE incidences for all patients receiving BRV (*n* = 77) and patients receiving placebo (*n* = 40) were 62.3% and 72.5%, respectively (Table S5). Incidences of drug‐related TEAEs were 35.1% and 35.0%, discontinuations due to TEAEs were 5.2% and 7.5%, serious TEAEs were 2.6% and 0, and severe TEAEs were 1.3% and 0 for all patients receiving BRV and for patients receiving placebo, respectively.

### 3.5 Post hoc analysis by most common concomitant ASMs

#### 3.5.1 Baseline demographics and epilepsy characteristics

The most common concomitant ASMs (≥30 patients) in patients randomized to BRV 50 mg/day and 200 mg/day were VPA, CBZ, and LTG (Table 2). Patients randomized to placebo or BRV on concomitant CBZ (*n* = 136) were younger at age of epilepsy onset (mean [SD] age, 14.5 [10.4] years) versus those on LTG and VPA (*n* = 100 and *n* = 177; 17.8 [13.6] and 18.4 [12.8] years, respectively) and had a longer duration of epilepsy (mean [SD] duration, 22.0 [13.3] years) vs. those on LTG and VPA (17.4 [12.2] and 16.1 [12.5] years, respectively). Patients on concomitant LTG had higher baseline median 28‐day focal‐onset seizure frequency (10.5) versus those on CBZ and VPA (8.2 and 7.1, respectively) and there was a lower proportion of patients with 0–1 previous ASMs (32 patients [32.0%]) versus those on CBZ and VPA (66 patients [48.5%] and 113 patients [63.8%], respectively).

#### 3.5.2 Efficacy

**FIGURE 3:**
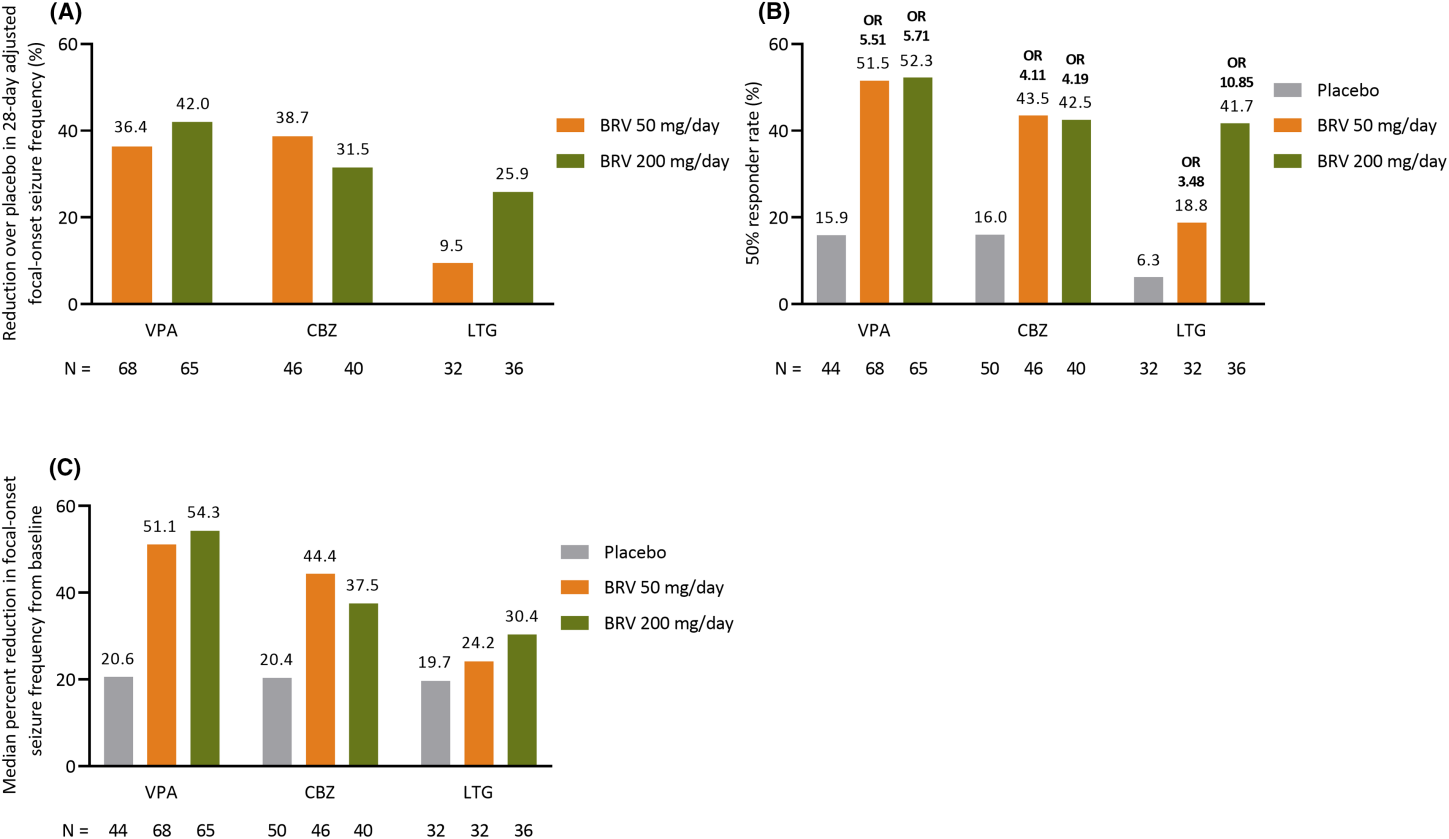
Efficacy for the post hoc analysis by common concomitant ASMs: (A) percent reduction over placebo in 28‐day adjusted focal‐onset seizure frequency, (B) 50% responder rate in focal‐onset seizure frequency from baseline^a^, and (C) median percent reduction in focal‐onset seizure frequency from basline (FAS). ^a^Odds ratios represent BRV versus placebo. ASM, antiseizure medication; BRV, brivaracetam; CBZ, carbamazepine; FAS, full analysis set; LTG, lamotrigine; OR, odds ratio; VPA, valproate.

BRV provided clinically relevant percent reductions over placebo in 28‐day adjusted focal‐onset seizure frequency, irrespective of the type of most common concomitant ASM used (Figure 3A). The 50% responder rate for focal‐onset seizure frequency was greater with BRV versus placebo in patients on concomitant VPA, CBZ, or LTG. A numerically higher response was observed in patients on concomitant VPA (Figure 3B). Treatment with BRV also resulted in greater median percent reductions from baseline in 28‐day adjusted focal‐onset seizure frequency than placebo, irrespective of whether patients were taking concomitant VPA, CBZ, or LTG (Figure 3C).

#### 3.5.3 Safety and tolerability

In patients on VPA, TEAE incidences for all patients receiving BRV (*n* = 133) and patients receiving placebo (*n* = 44) were 53.4% and 52.3%, respectively (Table S6). Incidences of drug‐related TEAEs were 27.1% and 13.6%, and discontinuations due to TEAEs were 3.0% and 2.3% for all patients receiving BRV and patients receiving placebo, respectively. No serious or severe TEAEs were reported.

In patients on CBZ, TEAE incidences for all patients receiving BRV (*n* = 86) and patients receiving placebo (*n* = 52) were 61.6% and 69.2%, respectively (Table S6). Incidences of drug‐related TEAEs were 36.0% and 21.2%, discontinuations due to TEAEs were 3.5% and 7.7%, serious TEAEs were 4.7% and 1.9%, and severe TEAEs were 1.2% and 1.9% for all patients receiving BRV and patients receiving placebo, respectively.

In patients on LTG, TEAE incidences for all patients receiving BRV (*n* = 68) and patients receiving placebo (*n* = 33) were 69.1% and 54.5%, respectively (Table S6). Incidences of drug‐related TEAEs were 39.7% and 18.2%, discontinuations due to TEAEs were 2.9% and 3.0%, serious TEAEs were 1.5% and 3.0%, and severe TEAEs were 0 and 3.0% for all patients receiving BRV and patients receiving placebo, respectively.

## 4 DISCUSSION

This phase III study evaluated efficacy, safety, and tolerability of BRV in a randomized, double‐blind, placebo‐controlled, multicenter setting in Asian patients aged ≥16–80 years with focal‐onset seizures with or without secondary generalization.

The study had a high completion rate (425 patients [94.7%]), with low rates of discontinuation of BRV across both BRV treatment groups (3.3% and 5.4% in the BRV 50 mg/day and BRV 200 mg/day groups, respectively) and low, but numerically higher, rates of discontinuation in patients receiving placebo (7.4%). All treatment groups were well balanced with respect to demographic and baseline disease characteristics.

The primary efficacy endpoint (percent reduction over placebo in 28‐day focal‐onset seizure frequency over the 12‐week treatment period) was statistically significant for both BRV treatment groups. The efficacy response seen in this trial is consistent with previous phase III fixed‐dose studies of BRV.^8^, ^9^, ^10^, ^11^, ^12^, ^13^ Percent reduction over placebo in 28‐day focal‐onset seizure frequency for the BRV 200 mg/day dose in this study (33.4%) was numerically higher than was observed in the phase III international N01358 study (23.2%), although this comparison is not straightforward due to the different patient populations studied.^9^ Secondary efficacy endpoints were supportive of BRV efficacy, again showing statistical significance of the two BRV treatment groups versus the placebo group.

Of note, a high placebo response was observed in this study. The causes of a placebo response are complex and multifactorial.^27^ A meta‐analysis of 33 clinical trials involving five different ASMs demonstrated that the placebo response in East Asian trials was statistically higher than that in Western trials, although the reasons for this difference between geographic locations were unclear.^28^ Other factors that could potentially affect placebo response are patient age at diagnosis and age at exposure to placebo,^27^ patient‐specific disease characteristics (e.g., disease duration,^28^, ^29^ disease classification,^28^, ^29^ ASM treatment history^27^, ^28^, ^29^), and year of clinical trial data publication.^28^, ^30^

BRV was well tolerated at the doses evaluated. Incidences of TEAEs were generally similar across patients randomized to BRV or placebo. The most common nonserious TEAEs were similar to those observed in previous BRV studies, as were incidences of serious TEAEs.^8^, ^9^, ^10^, ^11^, ^12^, ^13^ No unexpected TEAEs occurred. Incidences of psychiatric TEAEs including irritability were low in patients randomized to BRV or placebo and consistent with previous findings.^31^, ^32^ There was no clinically significant effect of BRV on laboratory parameters, vital signs and body weight, ECG evaluations, physical/neurological examinations, or mental/psychiatric status.

Pharmacokinetic evaluations showed plasma concentrations of BRV were consistent throughout the treatment period, indicating consistent absorption and a dose‐proportional increase in BRV plasma concentration similar to that of non‐Asian patients observed in a previous study.^33^

Results of the subgroup analyses were generally consistent with the overall population. Adjunctive BRV was generally well tolerated in both LEV‐naïve patients and those with previous LEV use, as well as when combined with commonly used concomitant ASMs. LEV‐naïve patients had a numerically greater efficacy response to BRV treatment than those with previous LEV use; however, the difference in response was small, and clinically relevant efficacy was shown in patients who had previously failed LEV as shown by the 50% responder rate odds ratios. This is consistent with previous studies and provides further evidence that previous treatment failure with LEV does not preclude the use of BRV, which has up to a 20‐fold higher affinity for SV2A versus LEV.^9^, ^21^ Real‐world evidence has also shown that add‐on BRV improved seizure control in LEV‐naïve patients and in patients that previously used LEV.^16^ Adjunctive BRV also showed efficacy in patients on concomitant VPA, CBZ, or LTG, the three most common concomitant ASMs in this study, which are frequently prescribed in clinical practice. This aligns with other BRV studies that have shown BRV to be efficacious and well tolerated, independent of the type or number of concomitant ASMs.^22^ This includes concomitant ASMs with other mechanisms of action, including sodium channel blockade and inhibition of GABA transaminase.^34^

Although outside the scope of the current analysis, topics that can be explored in future studies include the time course and sustainability of efficacy outcomes,^35^, ^36^ efficacy and tolerability in the elderly,^37^ efficacy in patients with very active focal epilepsy,^38^ and BRV outcomes stratified by reason of previous LEV discontinuation.

### 4.1 Study limitations

The subgroup analysis by LEV status was not further stratified by reason for previous LEV failure; therefore, it is unknown whether the responses observed differed between patients who discontinued LEV due to issues with inadequate efficacy versus tolerability. Results of the subgroup analysis should be interpreted with caution; sample size varied across subgroups analyzed and was relatively small in some subgroups.

## 5 Conclusions

Adjunctive BRV initiated without titration was efficacious and well tolerated in Asian patients with focal‐onset seizures. Reduction in 28‐day focal seizure frequency over placebo was statistically significant for the BRV 50 mg/day and 200 mg/day groups. BRV was also efficacious in patients on other commonly prescribed ASMs such as VPA, CBZ, and LTG. Incidences of TEAEs, TEAEs leading to discontinuation, and serious TEAEs were similar in patients randomized to placebo or BRV, showing that patients can start BRV treatment at therapeutic doses. No unexpected TEAEs occurred. Efficacy and safety/tolerability profiles were consistent with BRV studies in predominantly non‐Asian populations.

## AUTHOR CONTRIBUTIONS

**Yushi Inoue**: Methodology (equal), Investigation (equal), Writing—Review and Editing (equal). **Somsak Tiamkao**: Investigation (equal), Writing—Review and Editing (equal). **Dong Zhou**: Investigation (equal), Writing—Review and Editing (equal). **Leonor Cabral‐Lim**: Investigation (equal), Writing—Review and Editing (equal). **Kheng Seang Lim**: Investigation (equal), Writing—Review and Editing (equal). **Shih‐Hui Lim**: Investigation (equal), Writing—Review and Editing (equal). **Jing‐Jane Tsai**: Investigation (equal), Writing—Review and Editing (equal). **Brian Moseley**: Visualization (equal), Project Administration (lead), Supervision (lead), Formal Analysis (equal), Writing—Review and Editing (equal). **Lin Wang**: Writing—Review and Editing (equal). **Weiwei Sun**: Formal Analysis (equal), Writing—Review and Editing (equal). **Yoshinobu Hayakawa**: Writing—Review and Editing (equal). **Hiroshi Sasamoto**: Writing—Review and Editing (equal). **Tomonobu Sano**: Conceptualization (lead), Methodology (equal), Writing—Review and Editing (equal). **Carrie McClung**: Writing—Review and Editing (equal). **Almasa Bass**: Conceptualization (supporting), Supervision (lead), Writing—Review and Editing (equal).

## FUNDING INFORMATION

This study was funded by UCB Pharma, which was involved in the design of the study; the collection, analysis, and interpretation of data; and the decision to publish the manuscript.

## CONFLICT OF INTEREST STATEMENT

YI acts as a consultant for Eisai and UCB Pharma. DZ is the Deputy Editor for Epilepsia Open. ST, LC‐L, KSL, S‐H L, and JJT declare no conflicts of interest. BM, LW, WS, YH, HS, TS, CM, and AB are employees of UCB Pharma. BM, HS, and TS have stocks or stock options in UCB Pharma. We confirm that we have read the Journal's position on issues involved in ethical publication and affirm that this report is consistent with those guidelines.

## PATIENT CONSENT STATEMENT

Patient informed consent was approved by local institutional review boards/independent ethics committees, as defined in local regulations. Patients provided informed consent or had a legally authorized representative sign the informed consent on their behalf before completing any study‐related procedures.

## Supporting information


Appendix S1

Figure S1.

Appendix S2

Table S1

## Data Availability

Underlying data from this manuscript may be requested by qualified researchers 6 months after product approval in the U.S. and/or Europe, or global development is discontinued, and 18 months after trial completion. Investigators may request access to anonymized individual patient‐level data and redacted trial documents, which may include analysis‐ready data sets, study protocol, annotated case report forms, statistical analysis plans, data set specifications, and clinical study reports. Prior to use of the data, proposals need to be approved by an independent review panel at www.Vivli.org and a signed data‐sharing agreement will need to be executed. All documents are available in English only, for a prespecified time, typically 12 months, on a password‐protected portal.
