# Supplementary material for: Efficacy, safety, and tolerability of adjunctive brivaracetam in adult Asian patients with uncontrolled focal‐onset seizures: A phase III randomized, double‐blind, placebo‐controlled trial
Source: Epilepsia Open. 2024 Apr 4;9(3):1007–20. doi: 10.1002/epi4.12929 (PMC11145603; doi:10.1002/epi4.12929)
Supplement: Supplementary file 1 — Appendix S1 [file EPI4-9-1007-s004.pdf]

Efficacy, safety, and tolerability of adjunctive brivaracetam in adult Asian patients with uncontrolled focal-onset seizures: A phase III randomized, double-blind, placebo-controlled trial

Yushi Inoue │ Somsak Tiamkao │ Dong Zhou │ Leonor Cabral-Lim │ Kheng Seang Lim │ Shih-Hui Lim │ Jing-Jane Tsai │ Brian Moseley │ Lin Wang │ Weiwei Sun │ Yoshinobu Hayakawa │ Hiroshi Sasamoto │ Tomonobu Sano │ Carrie McClung │ Almasa Bass

List of investigators

The authors acknowledge the EP0083 trial investigators for their contributions to data acquisition: Aihua Liu, Neurology (Xuanwu Hospital, Capital Medical University, Beijing, China); Akihiro Shimotake, Neurology (Kyoto University Hospital, Kyoto, Japan); Akihiro Yasuhara, Pediatrics (Yasuhara Children's Clinic, Osaka, Japan); Annabelle Lao-Reyes, MD, Neurology (Metro Davao Medical and Research Center, Davao City, Philippines); Ayataka Fujimoto, Comprehensive Epilepsy Center (Seirei Hamamatsu General Hospital, Shizuoka, Japan); Bing Qin, Neurology (The First Affiliated Hospital of Jinan University, Guangzhou, Guangdong, China); Chikako Kaneko, Neurology (Southern Tohoku Medical Clinic, Fukushima, Japan); Chizuru Ikeda, Pediatrics (Kumamoto Saishunso National Hospital, Kumamoto, Japan); Chou-Ching Lin, MD, Neurology (National Cheng Kung University Hospital, Tainan, Taiwan); Chung-Yao Hsu, MD, Neurology (Kaohsiung Medical University - Chung-Ho Memorial Hospital, Kaohsiung City Kaohsiung Special Municipality, Taiwan); Chusak Limotai, MD, Neurology (King Chulalongkorn Memorial Hospital, Bangkok, Thailand); Eiichi Ishikawa, Neurosurgery (University of Tsukuba Hospital, Ibaraki, Japan); Fei Yi, Neurology (PingXiang People's Hospital, Pingxiang, China); Gaik Bee Eow, MD, Neurology (Hospital Pulau Pinang, Pulau Pinang, Malaysia); Haruhiko Kishima, Neurosurgery (Osaka University Hospital, Osaka, Japan); Hideaki Shiraishi, Pediatric (Hokkaido University Hospital, Hokkaido, Japan); Hidenori Sugano, Neurosurgery (Juntendo

University Hospital, Tokyo, Japan); Hidetoshi Nakamoto, Neurosurgery (TMG Asaka Medical Center, Asaka, Saitama, Japan); Hiromichi Motooka, Neurology (Kurume University Hospital, Fukuoka, Japan); Hirotomo Ninomiya, Neurosurgery (Itami City Hospital, Hyogo, Japan); Hisashi Okada, Neurology (Nagoya Medical Center, Aichi, Japan); Hui Liu, Neurology (ZhuJiang Hospital of Southern Medical University, Guangdong, China); Huiqin Xu, Neurology (The First Affiliated Hospital of Wenzhou Medical University, Zhejiang, China); Ichiro Takumi, Neurosurgery (St. Marianna University Hospital, Kanagawa, Japan); Irene Looi, MD, Neurology (Hospital Seberang Jaya, Pulau Pinang, Malaysia); Jinggui Song, Neurology (Henan Mental Diseases Hospital, Xinxiang City, Henan Province, China); Jinsheng Zeng, Neurology (The First Affiliated Hospital, Sun Yat-sen University, Guangdong, China); Jo Janette De La Calzada, MD, Neurology (Cebu Doctors University Hospital, Cebu City, Philippines); Joel Advincula, MD, Neurology (St. Paul's Hospital of Iloilo, Inc., Iloilo City, Philippines); Junya Kawada, Neurology (Shonan Kamakura General Hospital, Kanagawa, Japan); Kanoksri Samintarapanya, MD, Neurology (Lampang Hospital, Lampang, Thailand); Kanokwan Boonyapisit, MD, Neurology (Siriraj Hospital, Mahidol University, Bangkok, Thailand); Katsuhisa Uruno, Neurology (National Hospital Organization Yamagata National Hospital, Yamagata, Japan); Keisuke Toda, Neurosurgery (National Hospital Organization Nagasaki Kawatana Center, Nagasaki, Japan); Kheng Seang Lim, MD, Neurology (University Malaya Medical Center, Kuala Lumpur, Malaysia); Kiyohito Terada, Neurology (Yokohama Minoru Epilepsy & Developmental Clinic, Kanagawa, Japan); Koji Iida, Neurosurgery (Hiroshima University Hospital, Hiroshima, Japan); Kousuke Kanemoto, Neuropsychiatry (Aichi Medial University Hospital, Aichi, Japan); Leonor Cabral-Lim, MD, Neurology (Philippine General Hospital, Manila, Philippines); Margaret Sia-Modequillo, MD, Neurology (Perpetual Succour Hospital, Cebu City, Philippines); Maria Estrella Ibe, MD, Neurology (Philippine Heart Center, Quezon City, Philippines); Marissa Lukban, MD, Neurology (Manila Doctors Hospital, Manila, Philippines); Masafumi Fukuda, Functional Neurosurgery (Nishi-Niigata Chuo National Hospital, Niigata, Japan); Masaki Iwasaki, Neurosurgery (National Center of Neurology and Psychiatry, Tokyo, Japan); Masaki Tanaka,

Pediatrics (Tanaka Neurology Clinic, Kanagawa, Japan); Masako Kinoshita, Neurology (Utano National Hospital, Kyoto, Japan); Masako Watanabe, Psychiatry (Shinjuku Neuro Clinic, Tokyo, Japan); Misa Nakano, Neurology (Suita Municipal Hospital, Osaka, Japan); Naotaka Usui, Epilepsy (National Hospital Organization Shizuoka Institute of Epilepsy and Neurological Disorders, Shizuoka, Japan); Nobukazu Nakasato, Epilepsy (Tohoku University Hospital, Miyagi, Japan); Pasiri Sithinamsuwan, MD, Neurology (Phramongkutklao Hospital, Bangkok, Thailand); Peiyuan F. Hsieh, MD, Neurology (TaiChung Veterans General Hospital, Taichung, Taiwan); Qing Zhang, Neurology (General Hospital of Ningxia Medical University, Ningxia, China); Qun Wang, Neurology (Tiantan Hospital, Capital Medical University, Beijing, China); Rahul Rathakrishnan, MD, Neurology (National University Hospital, Singapore); Raymond Rosales, MD, Neurology (Metropolitan Medical Center, Manila, Philippines); Riki Matsumoto, Neurology (Kyoto University Hospital, Kyoto, Japan); Rose Izura Abdul Hamid, MD, Neurology (Hospital Raja Perempuan Zainab II, Kelantan, Malaysia); Sapiah Sapuan, MD, Neurology (Hospital Sungai Buloh Jalan Hospital, Selangor, Malaysia); Shigeya Tanaka, Neurosurgery (Tanaka Neurosurgical Clinic, Kagoshima, Japan); Shih-Hui Lim, MD, Neurology (Singapore General Hospital, Singapore); Shuang Wang, Neurology (The Second Affiliated Hospital of Zhejiang University, Hangzhou City, Zhejiang Province, China); Somsak Tiamkao, MD, Neurology (Srinagarind Hospital - Khon Kaen University, Khon Kaen, Thailand); Subsai Kongsaengdao, MD, Neurology (Rajavithi Hospital, Bangkok, Thailand); Sunao Kaneko, Psychiatry (Minato Hospital, Aomori, Japan); Surat Tanprawate, MD, Neurology (Maharaj Nakorn Chaing Mai Hospital, Chiang Mai, Thailand); Suwat Srisuwannanukorn, MD, Neurology (Navamindradhiraj University, Vajira hospital, Bangkok, Thailand); Takefumi Hitomi, Neurology (Kyoto University Hospital; Kyoto, Japan); Taketoshi Maehara, Neurosurgery (Tokyo Medical and Dental University Hospital; Tokyo, Japan); Takeyo Sakurai, Neurology (Sakurai Clinic, Shiga, Japan); Taku Ochiai, Neurosurgery (Ochiai Neurological Clinic, Saitama, Japan); Tateki Morikawa, Pediatrics (Morikawa Clinic, Aichi, Japan); Teiichi Onuma, Psychiatry (Musashino-Kokubunji Clinic, Tokyo, Japan); Tem Lom Fam, MD, Neurology (Hospital Miri - General Practice, Sarawak,

Malaysia); Tiancheng Wang, Neurology (Lanzhou University Second Hospital, City, Gansu Province, China); Toru Hoshida, Neurosurgery (Nara Medical Center, Nara, Japan); Wan Chung Law, MD, Neurology (Sarawak General Hospital, Kuching, Malaysia); Wangtao Zhong, Neurology (Affiliated Hospital of Guangdong Medical University, Guangdong, China); Weiping Liao, Neurology (The Second Affiliated Hospital of Guangzhou Medical University, Guangdong, China); Weiping Wang, Neurology (The Second Hospital of Hebei Medical University, Shijiazhuang City, Hebei Province, China); Xiaohong Zhou, Neurology (Guangdong General Hospital, Guangdong, China); Xintong Wu, Neurology (West China Hospital of Sichuan University, Chengdu City, Sichuan Province, China); Xiong Han, Neurology (Henan Provincial People’s Hospital, Zhengzhou City, Henan Province, China); Xufang Xie, Neurology (The First Affiliated Hospital of Nanchang University, Jiangxi, China); Yasuko Sawai, Neurosurgery (Nara Medical Center, Nara, Japan); Yasumichi Koide, Neurology (Koide Clinic of Epilepsy and Neurological Disorders, Osaka, Japan); Yasuo Iwadate, Neurosurgery (Chiba University Hospital, Chiba, Japan); Yi-Ting Hsu, MD, Neurology (China Medical University Hospital, Taichung, Taiwan); Yongping Dai, Neurology (The Second Affiliated Hospital of Soochow University, Jiangsu, China); Yu Koike, Neurology (Oasis Clinic, Ibaraki, Japan); Yuichi Kubota, Neurosurgery (Tokyo Women's Medical University Adachi Medical Center, Saitama, Japan); Yuping Wang, Neurology (Xuanwu Hospital, Capital Medical University, Beijing, China); Yusuke Wakita, Psychiatry (Minato Hospital, Aomori, Japan); Zariah Abdul Aziz, MD, Neurology (Hospital Sultanah Nur Zahirah, Terengganu, Malaysia); Ze Li, Neurology (Guangzhou First People’s Hospital; Guangdong, China); Zhiyong Chen, Neurology (The First Affiliated Hospital of Jinan University, Guangdong, China); Zucai Xu, Neurology (Affiliated Hospital of Zunyi Medical University, Zunyi City, Guizhou Province, China).
