# Supplementary material for: Efficacy, safety, and tolerability of adjunctive brivaracetam in adult Asian patients with uncontrolled focal‐onset seizures: A phase III randomized, double‐blind, placebo‐controlled trial
Source: Epilepsia Open. 2024 Apr 4;9(3):1007–20. doi: 10.1002/epi4.12929 (PMC11145603; doi:10.1002/epi4.12929)
Supplement: Supplementary file 2 — Figure S1. [file EPI4-9-1007-s003.pdf]

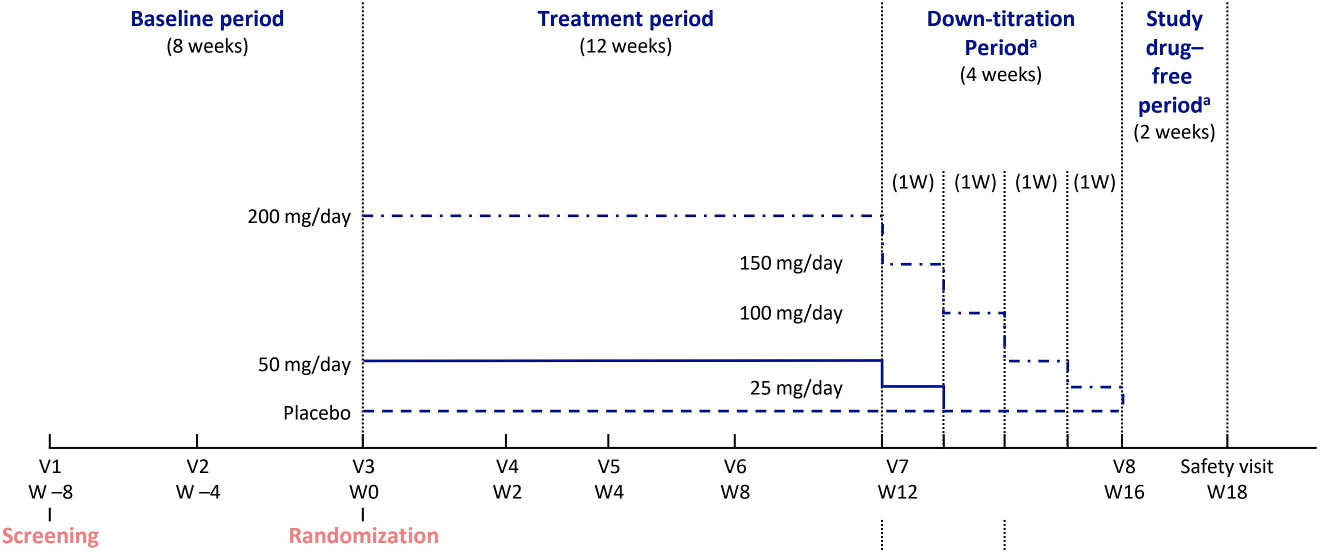

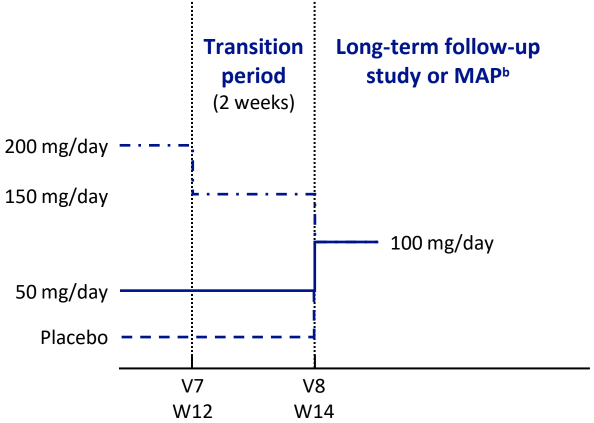

FIGURE S1. Study design.
[a]Study participants with an EDV at any time during the treatment period proceeded through the 4-week down-titration period and 2-week study drug–free period. [b]If the MAP was not ready at the end of the transition period, patients converted to the open-label temporary period for providing BRV.
Abbreviations: BRV, brivaracetam; EDV, early discontinuation visit; MAP, managed access program; V, visit; W, week.
