# Supplementary material for: Efficacy, safety, and tolerability of adjunctive brivaracetam in adult Asian patients with uncontrolled focal‐onset seizures: A phase III randomized, double‐blind, placebo‐controlled trial
Source: Epilepsia Open. 2024 Apr 4;9(3):1007–20. doi: 10.1002/epi4.12929 (PMC11145603; doi:10.1002/epi4.12929)
Supplement: Supplementary file 3 — Appendix S2 [file EPI4-9-1007-s002.pdf]

Efficacy, safety, and tolerability of adjunctive brivaracetam in adult Asian patients with uncontrolled focal-onset seizures: A phase III randomized, double-blind, placebo-controlled trial

Yushi Inoue | Somsak Tiamkao | Dong Zhou | Leonor Cabral-Lim | Kheng Seang Lim | Shih-Hui Lim | Jing-Jane Tsai | Brian Moseley | Lin Wang | Weiwei Sun | Yoshinobu Hayakawa | Hiroshi Sasamoto | Tomonobu Sano | Carrie McClung | Almasa Bass

Supplementary Methods

Full inclusion criteria

To be eligible to participate in this study, all the following criteria must have been met:

1. An institutional review board–/independent ethics committee–approved written informed consent form (ICF) was signed and dated by the study participant or by the parent(s) or legal representative. The ICF, or a specific assent form, where required, was signed and dated by minors.
2. Participant/legal representative was considered reliable and capable of adhering to the protocol (eg, able to understand and complete diaries), visit schedule, or investigational medicinal product (IMP) intake, according to the judgment of the investigator.
3. Participants (male or female) were aged 16–80 years at visit 1, both inclusive. Participants who were not legal adults were only included where legally permitted and ethically accepted.
4. Participants had a body weight ≥40 kg during the baseline period.
5. Female participants were without childbearing potential (postmenopausal for ≥2 years, bilateral oophorectomy or tubal ligation, complete hysterectomy). Female participants with childbearing potential were eligible if they used a medically accepted contraceptive method. Oral or depot contraceptive treatment with at least approved dosage of ethinylestradiol per intake in each country where this study was conducted (if participant

was taking any enzyme inducer such as carbamazepine, phenobarbital, primidone, phenytoin, oxcarbazepine, St. John's Wort, or rifampicin, the dosage of ethinylestradiol should have been modified according to the guidelines for contraceptive treatment in each country), monogamous relationship with vasectomized partner, or barrier contraception were acceptable methods. The participant must have understood the consequences and potential risks of inadequately protected sexual activity, must have been educated about and understood the proper use of contraceptive methods, and must have undertaken to inform the investigator of any potential change in status. Abstinence was considered as an acceptable method of contraception if the investigator could document that the participant agreed to be compliant.

6. Participant had well-characterized focal epilepsy/epileptic syndrome according to the 1989 International League Against Epilepsy (ILAE) classification.
7. Participant had an electroencephalogram (EEG) reading compatible with the clinical diagnosis of focal epilepsy within the last 5 years. Baseline EEG had to be scheduled and results received before visit 3 if no appropriate EEG was available within the last 5 years.
8. Participant had a brain magnetic resonance imaging (MRI)/computed tomography (CT) scan performed within the last 2 years. A CT scan or MRI had to be scheduled and results received before visit 3 if no previous CT scan or MRI was available within the last 2 years.
9. Participants had at least eight focal-onset (partial) seizures (according to the 1981 ILAE classification) during the 8-week baseline period, with at least two focal-onset (partial) seizures during each 4-week interval of the baseline period.
10. Participants had at least two focal-onset (partial) seizures, with or without secondary generalization, per month during the 3 months preceding visit 1.
11. Participant's condition was uncontrolled while treated by one or two permitted concomitant antiseizure medications (ASMs). Vagal nerve stimulation (VNS) was allowed and counted as a concomitant ASM.

12. Participant's permitted concomitant ASM(s) and VNS were stable and at optimal dosage for the participant for ≥4 weeks (12 weeks for phenobarbital, phenytoin, and primidone) before visit 1 and expected to be stable during whole study period.

Full exclusion criteria

Participants were not permitted to enroll in the study if they met any of the following criteria:

1. Participant was previously randomized within this study or any other previous study with brivaracetam (BRV) as a dosing arm.
2. Participant experienced simple focal-onset (partial) seizure (1981 ILAE classification) non-motor as the only seizure type.
3. Participant experienced febrile seizures exclusively. The occurrence of febrile seizures in addition to other unprovoked seizures was not exclusionary.
4. Participant had participated in another study of an investigational medicinal product (IMP) (or a medical device) within the last 30 days or was concurrently participating in another study of an IMP (or a medical device).
5. Participant was being concurrently treated with levetiracetam (LEV).
6. Participant had taken LEV within 90 days before visit 1.
7. Participant had any medical or psychiatric condition that, in the opinion of the investigator, could have jeopardized or would have compromised the participant's ability to participate in this study.
8. Participant had a known hypersensitivity to any components of the IMP, or any of its excipients.
9. Participant was not able to read and understand the ICF, assent form, or daily record card instructions.
10. Participant had severe cognitive impairment or mental retardation per investigator assessment.

11. Participant's seizures could not be reliably counted on a regular basis due to their fast and repetitive occurrence (clusters or flurries).
12. Participant had a history or presence of status epilepticus during the year preceding visit 1 or during baseline.
13. Participant had a history or presence of known psychogenic non-epileptic seizures.
14. Participant had taken vigabatrin for 24 weeks before visit 1. Participant with history of vigabatrin use, but either no visual field examination report was available, including standard static (Humphrey or Octopus) or kinetic perimetry (Goldman), or results of these examinations were abnormal, even though vigabatrin was stopped before 24 weeks.
15. Participant was taking any drug with possible central nervous system effects, except if stable for ≥4 weeks before visit 1 and was expected to be kept stable during whole study period.
16. Participant was taking any drug that significantly influenced the metabolism of BRV (cytochrome P450 strong inducers such as rifampin/rifampicin), except if the dose had been kept stable for ≥4 weeks before visit 1 and was expected to be kept stable during the treatment period.
17. Participant had a history of cerebrovascular accident, including transient ischemic attack, in the last 24 weeks.
18. Participant had severe cardiovascular disease or peripheral vascular disease.
19. Participant had the presence of any sign (clinical or imaging techniques) suggesting rapidly progressing (ie, not expected to stay stable during study participation) brain disorder or brain tumor. Stable arteriovenous malformations, meningiomas, or other benign tumors may have been acceptable.
20. Participant had any clinical conditions (eg, bone marrow suppression, severe renal impairment with creatinine clearance <30 mL/min, as calculated by the Cockcroft-Gault formula) that impaired reliable participation in the study or necessitated the use of IMP not allowed by protocol.

21. Participant had chronic hepatic disease.
22. Participant had the presence of a terminal illness.
23. Participant had the presence of a serious infection.
24. Participant had a history of severe adverse hematologic reaction to any drug.
25. Participant had severe disturbance of hemostasis.
26. Participant had >2× upper limit of normal (ULN) of any of the following: alanine aminotransferase (ALT), aspartate aminotransferase (AST), alkaline phosphatase (ALP), or >ULN total bilirubin (≥1.5× ULN total bilirubin if known Gilbert's syndrome). If participant had elevations only in total bilirubin that were >ULN and <1.5× ULN, fractionated bilirubin was to be used to identify possible undiagnosed Gilbert's syndrome (ie, direct bilirubin <35%). For randomized participants with a baseline result >ULN for ALT, AST, ALP, or total bilirubin, a baseline diagnosis and/or the cause of any clinically meaningful elevation must have been understood and recorded in the electronic case report form (eCRF). If participant had >ULN ALT, AST, or ALP that did not meet the exclusion limit at screening, the tests were to be repeated, if possible, before dosing, to ensure there was no further ongoing clinically relevant increase. In case of a clinically relevant increase, inclusion of the participant must have been discussed with the medical monitor.
27. Participant had a clinically significant laboratory abnormality that may have increased the risk associated with participation or may have interfered with the interpretation of study results, according to the judgment of the investigator.
28. Participant had clinically significant electrocardiogram abnormalities, according to the investigator.
29. Participant had a lifetime history of suicide attempt (including an active attempt, interrupted attempt, or aborted attempt), or had suicidal ideation in the past 6 months as indicated by a positive response ("yes") to either Question 4 or Question 5 of the Columbia-Suicide Severity Rating Scale at visit 1.
30. Participant had known multiple drug allergies or severe drug allergy.

31. Participant was pregnant or lactating.
32. Participant had a known alcohol or drug addiction or abuse within the last 2 years.
33. Participant was an investigator, co-investigator, their spouse or child, or any study collaborator. If the investigator had any other doubts concerning the eligibility, he/she was to consult the UCB study physician or a representative for clarification.

Sample size calculations

- The sample size assumed treatment differences of 0.221 and 0.285 in means on the log-transformed scale and a common standard deviation (SD) of 0.66. The estimates of 0.221 and 0.285 corresponded to reductions of 19.8% and 24.8% of BRV over placebo after back-transformation, respectively. The percent reduction of 24.8% was obtained from BRV 200 mg/day in the N01358 study (UCB Pharma, data on file) and assumed the LEV cap of 30% was reached overall. Based on a similar result from the integrated summary of efficacy and again assuming the LEV cap of 30% was reached overall, a 19.8% reduction of BRV 50 mg/day over placebo could be estimated. The SD of 0.66 was based on the observed SD from all treatment groups in N01358. The sample size calculation accounted for the correlation of the test statistics (same placebo group was compared with each BRV dose), as well as the planned Hochberg procedure.

Blinding

- For the purposes of blinding, BRV and matching placebo products (as well as their packaging) were kept identical in appearance. Kit numbers were allocated according to the package list generated by a validated program by UCB Pharma. This list is provided to the interactive voice response system (IVRS).
- The treatment randomization schedule was generated by UCB (or designee) in a manner that ensured that the study team remained blinded. The randomization schedule was maintained in a secure location until the study was unblinded for the final statistical analysis.

- All sponsors, investigator sites, and clinical research organization staff were blinded to the treatment code, with the following exceptions: sponsor personnel and subcontractors directly involved in the packaging of the IMP or in the management of the IVRS; sponsor patient safety (PS) staff who received separate access to the IVRS to meet their requirements for serious adverse event reporting to regulatory authorities; and central laboratory staff assaying the IMP (however, no randomization list was provided).

Stratification

- The number of participants for each stratification factor per the IVRS and eCRF is shown below:
  - Per the IVRS, 333 patients (74.2%) were LEV naïve and 116 (25.8%) were previous LEV users, whereas, per the eCRF, 332 patients (73.9%) were LEV naïve and 117 (26.1%) were previous LEV users.
  - Per the IVRS, 299 patients (66.6%) had used two or fewer ASMs and 150 (33.4%) had used more than two ASMs, whereas, per the eCRF, 296 patients (65.9%) had used two or fewer ASMs and 153 (34.1%) had used more than two ASMs.

Protocol amendments

- Two substantial protocol amendments occurred after trial commencement.
- One was dated February 1, 2019, at which point 158 patients had been enrolled. The changes included an increase in previous LEV use limitation from 20% to 30%.

One was dated January 10, 2020, at which point 272 patients had been enrolled. The changes included the following: total sample size reduced from 504 to 444 patients; the minimum required number of Japanese patients was reduced; China Mainland was added to the list of participating countries and regions; and updates to exclusion criteria (patient clinical conditions and patient clinical laboratory abnormalities).
