# Supplementary material for: Efficacy, safety, and tolerability of adjunctive brivaracetam in adult Asian patients with uncontrolled focal‐onset seizures: A phase III randomized, double‐blind, placebo‐controlled trial
Source: Epilepsia Open. 2024 Apr 4;9(3):1007–20. doi: 10.1002/epi4.12929 (PMC11145603; doi:10.1002/epi4.12929)
Supplement: Supplementary file 4 — Table S1 [file EPI4-9-1007-s001.pdf]

Efficacy, safety, and tolerability of adjunctive brivaracetam in adult Asian patients with uncontrolled focal-onset seizures: A phase III randomized, double-blind, placebo-controlled trial

Yushi Inoue | Somsak Tiamkao | Dong Zhou | Leonor Cabral-Lim | Kheng Seang Lim | Shih-Hui Lim | Jing-Jane Tsai | Brian Moseley | Lin Wang | Weiwei Sun | Yoshinobu Hayakawa | Hiroshi Sasamoto | Tomonobu Sano | Carrie McClung | Almasa Bass

TABLE S1. Patient disposition and discontinuations (RS).

**Disposition, *n* (%)**	**Placebo (*n* = 149)**	**BRV 50 mg/day (*n* = 152)**	**BRV 200 mg/day (*n* = 148)**	**BRV all (*n* = 300)**
Started	149 (100)	152 (100)	148 (100)	300 (100)
Completed	138 (92.6)	147 (96.7)	140 (94.6)	287 (95.7)
Discontinued	11 (7.4)	5 (3.3)	8 (5.4)	13 (4.3)
Adverse event	5 (3.4)	4 (2.6)	5 (3.4)	9 (3.0)
Withdrawal by participant	3 (2.0)	0	0	0
Other	1 (0.7)	1 (0.7)	0	1 (0.3)
Protocol violation	1 (0.7)	0	1 (0.7)	1 (0.3)
Lost to follow-up	1 (0.7)	0	1 (0.7)	1 (0.3)
Lack of efficacy	0	0	1 (0.7)	1 (0.3)

Abbreviations: BRV, brivaracetam; RS, randomized set.

TABLE S2. Overall sensitivity analysis of percent reduction over placebo for 28-day adjusted focal-onset seizure frequency according to IVRS stratification level (FAS).

	**Placebo (*n* = 147)**	**BRV 50 mg/day (*n* = 151)**	**BRV 200 mg/day (*n* = 148)**
Back-transformed LS means	9.3	6.7	5.8
Percent reduction over placebo		25.1	34.2
95% CI (LL–UL)		(12.3–36.1)	(22.8–43.9)
p-value[a]		.0004[c]	<.0001[c]
p-value[b]		.0004[c]	<.0001[c]
Non-parametric analysis			
p-value[a]		.0007[d]	<.0001[d]

Abbreviations: ASM, antiseizure medication; BRV, brivaracetam; CI, confidence interval; eCRF, electronic case report form; FAS, full analysis set; IVRS, interactive voice response system; LL, lower limit; LS, least squares; UL, upper limit.

Note: Parametric effect estimates and treatment group comparisons were based on analysis of covariance with log-transformed [log(x + 1)] treatment period 28-day adjusted focal-onset seizure frequency as the outcome and an effect for treatment, an effect for country, and an effect for the four combinations of stratification levels for number of previous ASMs and LEV status according to the eCRF data, and log-transformed 28-day adjusted baseline focal-onset seizure frequency as a continuous covariate. Non-parametric comparisons for each BRV treatment group vs placebo were obtained by applying a Mantel-Haenszel test to the residuals from the regression of the ranks of 28-day adjusted focal-onset seizure frequency for the treatment period vs the ranks of 28-day adjusted focal-onset seizure frequency for the baseline period.

[a]*p*-values not adjusted for multiplicity. [b]Multiplicity-adjusted *p*-values based on a Hochberg multiple comparison procedure. [c]Statistically significant with control of type I error rate based on a Hochberg multiple comparison procedure. [d]Statistically significant at a nominal two-sided .05 significance level.

TABLE S3. BRV plasma concentrations per time window after dosing, by dose and visit (PK-PPS).

	**Observed plasma concentration, μg/mL**											
	**BRV 50 mg/day**						**BRV 200 mg/day**					
	**geoMean (geoCV%)**						**geoMean (geoCV%)**					
	n	**>0–4 hours**	***n***	**>4–8 hours**	***n***	**>8 hours**	***n***	**>0–4 hours**	***n***	**>4–8 hours**	***n***	**>8 hours**
Visit 4 (week 2)	120	0.69 (95.6)	18	0.67 (38.6)	8	0.18 (979.4)	99	3.43 (42.5)	27	2.06 (334.4)	15	1.21 (100.1)
Visit 5 (week 4)	118	0.76 (88.8)	18	0.65 (39.2)	7	0.40 (54.7)	101	3.0 (120.2)	31	2.85 (43.2)	7	1.11 (74.4)
Visit 6 (week 8)	115	0.77 (87.3)	19	0.76 (34.0)	7	0.28 (10.9)	102	3.0 (127.7)	24	2.7 (54.9)	9	1.56 (67.7)
Visit 7 (week 12)	98	0.77 (89.5)	28	0.65 (39.4)	10	0.18 (579.5)	97	3.25 (121.1)	29	1.60 (838.9)	9	1.5 (45.4)

Abbreviations: BRV, brivaracetam; geoCV, geometric coefficient of variation; geoMean, geometric mean; PK-PPS, pharmacokinetic per-protocol set.

TABLE S4. Dose-normalized BRV plasma concentrations per time window after dosing, by dose and visit (PK-PPS).

	Dose-normalized plasma concentration, µg/mL/50 mg											
	BRV 50 mg/day						BRV 200 mg/day					
	geoMean (geoCV%)						geoMean (geoCV%)					
	n	>0–4 hours	*n*	>4–8 hours	*n*	>8 hours	*n*	>0–4 hours	*n*	>4–8 hours	*n*	>8 hours
Visit 4 (week 2)	120	0.69 (95.6)	18	0.67 (38.6)	8	0.18 (979.4)	99	0.86 (42.5)	27	0.52 (334.4)	15	0.30 (100.1)
Visit 5 (week 4)	118	0.76 (88.8)	18	0.65 (39.2)	7	0.40 (54.7)	101	0.75 (120.2)	31	0.71 (43.2)	7	0.28 (74.4)
Visit 6 (week 8)	115	0.77 (87.3)	19	0.76 (34.0)	7	0.28 (10.9)	102	0.75 (127.7)	24	0.68 (54.9)	9	0.39 (67.7)
Visit 7 (week 12)	98	0.77 (89.5)	28	0.65 (39.4)	10	0.18 (579.5)	97	0.81 (121.1)	29	0.40 (838.9)	9	0.38 (45.4)

Abbreviations: BRV, brivaracetam; geoCV, geometric coefficient of variation; geoMean, geometric mean; PK-PPS, pharmacokinetic per-protocol set.

TABLE S5. Incidence of TEAEs in the subgroup analysis by LEV status[a] (SS).

Patients, *n* (%)	LEV naïve				Previous LEV use			
	**Placebo (*n* = 109)**	**BRV 50 mg/day (*n* = 109)**	**BRV 200 mg/day (*n* = 113)**	**BRV all (*n* = 222)**	**Placebo (*n* = 40)**	**BRV 50 mg/day (*n* = 42)**	**BRV 200 mg/day (*n* = 35)**	**BRV all (*n* = 77)**
Any TEAEs	58 (53.2)	60 (55.0)	67 (59.3)	127 (57.2)	29 (72.5)	26 (61.9)	22 (62.9)	48 (62.3)
Serious TEAEs	1 (0.9)	1 (0.9)	3 (2.7)	4 (1.8)	0	1 (2.4)	1 (2.9)	2 (2.6)
Discontinuation due to TEAEs	4 (3.7)	2 (1.8)	3 (2.7)	5 (2.3)	3 (7.5)	2 (4.8)	2 (5.7)	4 (5.2)
Drug-related TEAEs	16 (14.7)	28 (25.7)	44 (38.9)	72 (32.4)	14 (35.0)	12 (28.6)	15 (42.9)	27 (35.1)
Severe TEAEs	1 (0.9)	1 (0.9)	1 (0.9)	2 (0.9)	0	0	1 (2.9)	1 (1.3)
Deaths (TEAEs leading to death)	0	1 (0.9)	0	1 (0.5)	0	0	0	0

Abbreviations: BRV, brivaracetam; LEV, levetiracetam; SS, safety set; TEAE, treatment-emergent adverse event.
[a]Inclusive of all study periods except open-label temporary period.

TABLE S6. Incidence of TEAEs in the post hoc analysis by most common concomitant ASMs[a] (SS).

Patients, *n* (%)	Concomitant VPA				Concomitant CBZ				Concomitant LTG			
	**Placebo (*n* = 44)**	**BRV 50 mg/day (*n* = 68)**	**BRV 200 mg/day (*n* = 65)**	**BRV all (*n* = 133)**	**Placebo (*n* = 52)**	**BRV 50 mg/day (*n* = 46)**	**BRV 200 mg/day (*n* = 40)**	**BRV all (*n* = 86)**	**Placebo (*n* = 33)**	**BRV 50 mg/day (*n* = 32)**	**BRV 200 mg/day (*n* = 36)**	**BRV all (*n* = 68)**
Any TEAEs	23 (52.3)	36 (52.9)	35 (53.8)	71 (53.4)	36 (69.2)	24 (52.2)	29 (72.5)	53 (61.6)	18 (54.5)	24 (75.0)	23 (63.9)	47 (69.1)
Serious TEAEs	0	0	0	0	1 (1.9)	1 (2.2)	3 (7.5)	4 (4.7)	1 (3.0)	0	1 (2.8)	1 (1.5)
Discontinuation due to TEAEs	1 (2.3)	2 (2.9)	2 (3.1)	4 (3.0)	4 (7.7)	2 (4.3)	1 (2.5)	3 (3.5)	1 (3.0)	1 (3.1)	1 (2.8)	2 (2.9)
Drug-related TEAEs	6 (13.6)	16 (23.5)	20 (30.8)	36 (27.1)	11 (21.2)	13 (28.3)	18 (45.0)	31 (36.0)	6 (18.2)	13 (40.6)	14 (38.9)	27 (39.7)
Severe TEAEs	0	0	0	0	1 (1.9)	0	1 (2.5)	1 (1.2)	1 (3.0)	0	0	0
Deaths (TEAEs leading to death)	0	0	0	0	0	0	0	0	0	0	0	0

Abbreviations: ASM, antiseizure medication; BRV, brivaracetam; CBZ, carbamazepine; LTG, lamotrigine; SS, safety set; TEAE, treatment-emergent adverse event; VPA, valproate.

[a]Inclusive of all study periods except open-label temporary period.
